# Supplementary material for: Development of an epilepsy self-management mobile health app framework: Content validity study results
Source: PLoS One. 2024 Jun 7;19(6):e0302844. doi: 10.1371/journal.pone.0302844 (PMC11161114; doi:10.1371/journal.pone.0302844)
Supplement: S1 Appendix — (DOCX) [file pone.0302844.s001.docx]

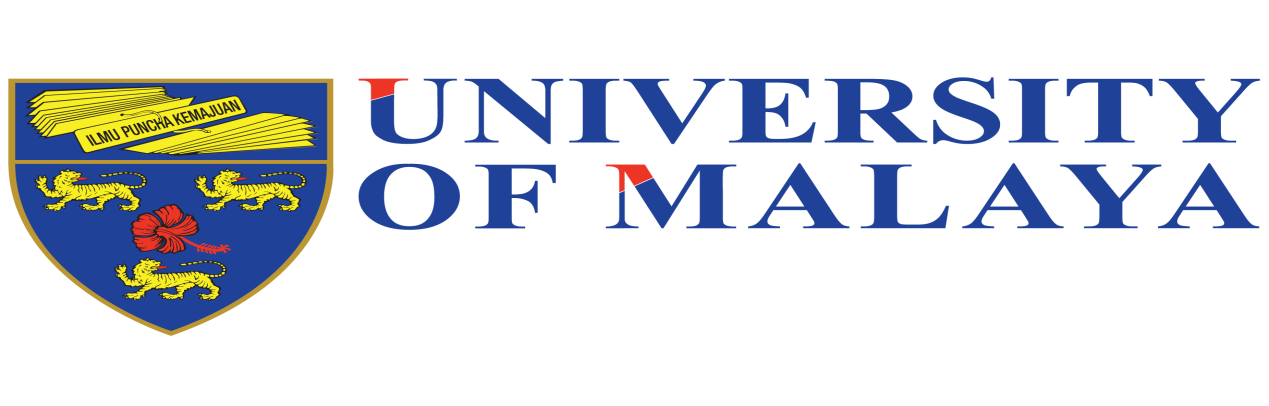


Dear:

| **Invitation as Expert Review Panel For Research Framework Validation** | |
| --- | --- |
| Covering  letter | **Invitation**  It is our pleasure to invite you to be one as an expert review panel to validate the mobile health application framework which enhance epilepsy self-management (eSM) to fulfill the research requirement. |
|  | **Purpose of research**  This research designed and developed a mobile health application framework which enhance the epilepsy self management rely on literature review and mobile apps review which are available in on the apps stores(iOS and Android)  The purpose of this study is to validate the proposal health mobile application  Framework that enhance epilepsy self management by experts panel review. |
|  | **Expectations from the reviewer.**  As an expert, I request you to help me to validate the following:   1. Items reflecting the Domain (stage one you need to assess the items which will be sent to you through email)   B) participate on group meeting (stage two interview meeting to get your comments and information that can help to improve the proposal framework) |
|  | **Instructions to the reviewer**  **Please be as objective and constructive as possible in your review and used the**  Use Relevance Scale to evaluate the items relate to eSM's domains also, participate in the meeting as experts.  You will get invitation through Doodle Pool to determine the preferable time you would like for the meeting. |
|  | It is hoped that your participation as an expert review panel will help to improve the framework. If you have any concerns about this please contact me at  Mohsen Alzamananan  [azayed2007@hotmail.com](mailto:azayed2007@hotmail.com).  Your kind cooperation and assistance in this regard is highly appreciated. |
