# Supplementary material for: Development of an epilepsy self-management mobile health app framework: Content validity study results
Source: PLoS One. 2024 Jun 7;19(6):e0302844. doi: 10.1371/journal.pone.0302844 (PMC11161114; doi:10.1371/journal.pone.0302844)
Supplement: S2 Appendix — (DOCX) [file pone.0302844.s002.docx]

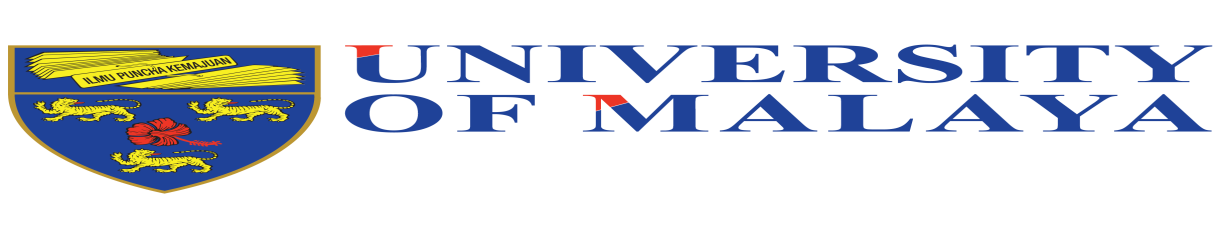


**Assessment Items Relate to Mobile Health Application Framework Which Enhance Epilepsy self-management**

| **Mobile Health Application Framework which enhance epilepsy self-management** | | | | | |
| --- | --- | --- | --- | --- | --- |
| **Expert Assessment**: 1.**The items is not relevant to measured domain 2.The item is somewhat relevant to the measured domain.3.The item is quite relevant to the measured domain. 4. The item is highly relevant to the measured domain.** | | | | | |
| **Domain's Definition** | **Items Reflect the Domains** | **Relevance** | | | |
|  |  | 1 | 2 | 3 | 4 |
| **Seizure tracking:** It provides critical information that helps the health care professional to evaluate the medication and health treatment received by the patients[[1](#_ENREF_1)] | 1. Keep tracking of seizures. | ⃝ | ⃝ | ⃝ | ⃝ |
|  | 2. Keep tracking of seizures occurring. | ⃝ | ⃝ | ⃝ | ⃝ |
|  | 3. Keep a recording of the types of seizures. | ⃝ | ⃝ | ⃝ | ⃝ |
|  | 4. Recognize situations that might cause seizures. | ⃝ | ⃝ | ⃝ | ⃝ |
|  | 5. Avoid situations or things that might cause seizures. | ⃝ | ⃝ | ⃝ | ⃝ |
|  | 6. Calling health care provider when having more of different seizure than usual. | ⃝ | ⃝ | ⃝ | ⃝ |
|  | Comments or suggestions. | | | | |
| **Medication Adherence:**  Nonadherence is a critical issue for PWEs[[2](#_ENREF_2)] and can lead to an increase in seizure frequency[[3](#_ENREF_3)]. PWEs generally do not adhere to their medication, which negatively affects their situation[[4](#_ENREF_4)]  Nonadherence to medications usually decreases the quality of treatment outcomes, maximizes the consultations and hospitalization, and increases the health care cost[[5](#_ENREF_5)] | 1. Having a way to remind to take seizure medicine. | ⃝ | ⃝ | ⃝ | ⃝ |
|  | 2. Taking seizure medicine as prescribed even on holidays, birthdays. Vacations, and other special occasions. | ⃝ | ⃝ | ⃝ | ⃝ |
|  | 3. Taking seizure medicine at about the same time each day. | ⃝ | ⃝ | ⃝ | ⃝ |
|  | 4. Taking seizure medicine the way health-care provider prescribes it. | ⃝ | ⃝ | ⃝ | ⃝ |
|  | Comments and suggestions. | | | | |
| **Treatment Management:**  It is a process which help patient to reminder for treatment, appointment, and recommendations and advice of HCPs that should be followed[[6](#_ENREF_6)] | 1. Keeping health-care provider or clinic appointments | ⃝ | ⃝ | ⃝ | ⃝ |
|  | 2. Having ways to remember things that have to do. | ⃝ | ⃝ | ⃝ | ⃝ |
|  | 3. Before seizure medicine runs out, get it refilled. | ⃝ | ⃝ | ⃝ | ⃝ |
|  | 4. Having blood tests or other tests done when ordered by health-care provider. | ⃝ | ⃝ | ⃝ | ⃝ |
|  | 5. Adjust or change medication when it causes side effect through call without visit my doctors.(self-developed) | ⃝ | ⃝ | ⃝ | ⃝ |
|  | 6.Talking with someone about epilepsy/seizure when need to | ⃝ | ⃝ | ⃝ | ⃝ |
|  | Suggestion and comments | | | | |
| **Health Care Communication:**  The success of the patient-centered model depends on communication between HCPs and patients[[7](#_ENREF_7)] which strengthens their relationship and facilitates better treatment. | 1. Talk with HCP about the long term effect of epilepsy treatment. | ⃝ | ⃝ | ⃝ | ⃝ |
|  | 2.Talk about how take seizure medicine with HCP | ⃝ | ⃝ | ⃝ | ⃝ |
|  | 3.Talk with HCP about sleep habits | ⃝ | ⃝ | ⃝ | ⃝ |
|  | 4.Send weekly, monthly report through what’s up to HCP (self-developed) | ⃝ | ⃝ | ⃝ | ⃝ |
|  | 5. Talk to health-care provider about emotions. | ⃝ | ⃝ | ⃝ | ⃝ |
|  | Comments or suggestions | | | | |

**Demographic variables**

**Part A: Please choose your more suitable answer.**

1. What is your age?

- 19 or younger
- 20 to 29
- 30 to 39
- 40 or older

1. What is your gender?

- Female
- Male

1. What is your Occupation

- Doctor
- Nurse
- Other …………

1. What is your level of education?

- Elementary / Primary education
- High school diploma
- Bachelor's degree
- Master's degree
- Ph.D. / Doctorate

1. How long is your experience?

- More than 2 years
- Between 5-8 years
- 10 to 15 year
- More than 15 years
- Other:

**References**

1. Rich, A., et al., *Theory of planned behavior and adherence in chronic illness: a meta-analysis.* Journal of behavioral medicine, 2015. **38**(4): p. 673-688.

2. Escoffery, C., et al., *Development of the adult epilepsy self-management measurement instrument(AESMMI).* Epilepsy & Behavior, 2015. **50**: p. 172-183.

3. Bidwell, J., et al., *Seizure reporting technologies for epilepsy treatment: a review of clinical information needs and supporting technologies.* Seizure, 2015. **32**: p. 109-117.

4. Lua, P.L. and W.S. Neni, *Feasibility and acceptability of mobile epilepsy educational system (MEES) for people with epilepsy in Malaysia.* TELEMEDICINE and e-HEALTH, 2012. **18**(10): p. 777-784.

5. Lund, A.M., *Measuring usability with the use questionnaire12.* Usability interface, 2001. **8**(2): p. 3-6.

6. Demonceau, J., et al., *Identification and assessment of adherence-enhancing interventions in studies assessing medication adherence through electronically compiled drug dosing histories: a systematic literature review and meta-analysis.* Drugs, 2013. **73**(6): p. 545-562.

7. Alzamanan, M.Z., et al., *Self-management apps for people with epilepsy: systematic analysis.* JMIR mHealth and uHealth, 2021. **9**(5): p. e22489.
