# Supplementary material for: Development of an epilepsy self-management mobile health app framework: Content validity study results
Source: PLoS One. 2024 Jun 7;19(6):e0302844. doi: 10.1371/journal.pone.0302844 (PMC11161114; doi:10.1371/journal.pone.0302844)
Supplement: Attachment — Submitted filename: Plose one Reviewers comments_Edited.docx [file pone.0302844.s003.docx]

**View Letter**

| **Date:** | Mar 04 2024 10:14PM |
| --- | --- |
| **To:** | "Mohsen Zaied Alzamanan" azayed2007@hotmail.com |
| **From:** | "PLOS ONE" plosone@plos.org |
| **Subject:** | PLOS ONE Decision: Revision required [PONE-D-23-41088] |

PONE-D-23-41088

Development of an Epilepsy Self-Management Mobile Health App Framework

PLOS ONE

Dear Dr. Alzamanan,

Thank you for submitting your manuscript to PLOS ONE. After careful consideration, we feel that it has merit but does not fully meet PLOS ONE&rsquo;s publication criteria as it currently stands. Therefore, we invite you to submit a revised version of the manuscript that addresses the points raised during the review process.
This manuscript needs further substantial work; both the reviewers have raised serious concerns about the framework and the statistical part, as well as the references, to name a few. You are advised to carefully make changes suggested by the experts.

Please submit your revised manuscript by Apr 18 2024 11:59PM. If you will need more time than this to complete your revisions, please reply to this message or contact the journal office at [plosone@plos.org](mailto:plosone@plos.org). When you&#39;re ready to submit your revision, log on to https://www.editorialmanager.com/pone/ and select the &#39;Submissions Needing Revision&#39; folder to locate your manuscript file.

Please include the following items when submitting your revised manuscript:

- A rebuttal letter that responds to each point raised by the academic editor and reviewer(s). You should upload this letter as a separate file labeled &#39;Response to Reviewers&#39;.
- A marked-up copy of your manuscript that highlights changes made to the original version. You should upload this as a separate file labeled &#39;Revised Manuscript with Track Changes&#39;.
- An unmarked version of your revised paper without tracked changes. You should upload this as a separate file labeled &#39;Manuscript&#39;.

If you would like to make changes to your financial disclosure, please include your updated statement in your cover letter. Guidelines for resubmitting your figure files are available below the reviewer comments at the end of this letter.

If applicable, we recommend that you deposit your laboratory protocols in protocols.io to enhance the reproducibility of your results. Protocols.io assigns your protocol its own identifier (DOI) so that it can be cited independently in the future. For instructions see: <https://journals.plos.org/plosone/s/submission-guidelines#loc-laboratory-protocols>. Additionally, PLOS ONE offers an option for publishing peer-reviewed Lab Protocol articles, which describe protocols hosted on protocols.io. Read more information on sharing protocols at [https://plos.org/protocols?utm_medium=editorial-email&amp;utm_source=authorletters&amp;utm_campaign=protocols](https://plos.org/protocols?utm_medium=editorial-email&utm_source=authorletters&utm_campaign=protocols).

We look forward to receiving your revised manuscript.

Kind regards,

Muhammad Farooq Umer, PhD Epidemiology and Health Statistics
Academic Editor
PLOS ONE

Journal requirements:

1. When submitting your revision, we need you to address these additional requirements.

Please ensure that your manuscript meets PLOS ONE&#39;s style requirements, including those for file naming. The PLOS ONE style templates can be found at 
https://journals.plos.org/plosone/s/file?id=wjVg/PLOSOne_formatting_sample_main_body.pdf and 
https://journals.plos.org/plosone/s/file?id=ba62/PLOSOne_formatting_sample_title_authors_affiliations.pdf.

2. Please note that PLOS ONE has specific guidelines on code sharing for submissions in which author-generated code underpins the findings in the manuscript. In these cases, all author-generated code must be made available without restrictions upon publication of the work. Please review our guidelines at https://journals.plos.org/plosone/s/materials-and-software-sharing#loc-sharing-code and ensure that your code is shared in a way that follows best practice and facilitates reproducibility and reuse.

3. Please amend either the title on the online submission form (via Edit Submission) or the title in the manuscript so that they are identical.

4. Please include captions for your Supporting Information files at the end of your manuscript, and update any in-text citations to match accordingly. Please see our Supporting Information guidelines for more information: http://journals.plos.org/plosone/s/supporting-information.

[Note: HTML markup is below. Please do not edit.]

Reviewers&#39; comments:

Reviewer&#39;s Responses to Questions

**Comments to the Author**

1. Is the manuscript technically sound, and do the data support the conclusions?

The manuscript must describe a technically sound piece of scientific research with data that supports the conclusions. Experiments must have been conducted rigorously, with appropriate controls, replication, and sample sizes. The conclusions must be drawn appropriately based on the data presented.

Reviewer #1: Yes

Reviewer #2: Partly

2. Has the statistical analysis been performed appropriately and rigorously?

Reviewer #1: I Don&#39;t Know

Reviewer #2: N/A

3. Have the authors made all data underlying the findings in their manuscript fully available?

The [PLOS Data policy](http://www.plosone.org/static/policies.action#sharing) requires authors to make all data underlying the findings described in their manuscript fully available without restriction, with rare exception (please refer to the Data Availability Statement in the manuscript PDF file). The data should be provided as part of the manuscript or its supporting information, or deposited to a public repository. For example, in addition to summary statistics, the data points behind means, medians and variance measures should be available. If there are restrictions on publicly sharing data&mdash;e.g. participant privacy or use of data from a third party&mdash;those must be specified.

Reviewer #1: Yes

Reviewer #2: Yes

4. Is the manuscript presented in an intelligible fashion and written in standard English?

PLOS ONE does not copyedit accepted manuscripts, so the language in submitted articles must be clear, correct, and unambiguous. Any typographical or grammatical errors should be corrected at revision, so please note any specific errors here.

Reviewer #1: No

Reviewer #2: Yes

5. Review Comments to the Author

Please use the space provided to explain your answers to the questions above. You may also include additional comments for the author, including concerns about dual publication, research ethics, or publication ethics. (Please upload your review as an attachment if it exceeds 20,000 characters)

Reviewer #1: This study aimed to assess the practicality and usability of the proposed mHealth app framework designed to enhance Epilepsy Self-Management Mobile Health App through an expert panel review. Thank you for the opportunity reviewing this article. I may require some comments on the following issues.
Abstract:
The findings section is long, in this section only mention the main results.

Thank you. We have now shortened this section.

Check keywords with mesh to make sure they are standard.
Introduction:
The introduction section should be made stronger.

Thank you. We have amended the section and all new changes are highlighted.

In the second or third paragraph of this section, mention the importance of m-health in the management of chronic conditions. For this purpose, use the following related articles.
https://pubmed.ncbi.nlm.nih.gov/29726434/
https://pubmed.ncbi.nlm.nih.gov/28508813/
https://pubmed.ncbi.nlm.nih.gov/29726430/

Methods:
the following structure should be observed for the method part:
1. Study Design and Search Strategy
2. Data sources
3. Study selection
4. Data extraction
5. Quality and risk of bias assessment

Thank you. This has been structured as applicable.
Discussion:
There is considerable gap in discussion section relating to discuss about your findings using related studies. So, use the following articles this section as well.
https://www.sciencedirect.com/science/article/abs/pii/S2211883720300642
https://pubmed.ncbi.nlm.nih.gov/33227362/
https://pubmed.ncbi.nlm.nih.gov/34723447/
Please include limitations section within the discussion section (preferably last paragraph).

Thank you so much for sharing these articles. These certainly helped us improve the “Discussion” section. Further, we have also included our study limitations to the “Discussion” section as suggested.

Reviewer #2: Developing an Epilepsy Self-Management Mobile Health App Framework is worthy work on the subject; however, the manuscripts lack crucial and fundamental concepts.

1- I’m concerned that the authors overlooked any theories considered when developing the Epilepsy Self-Management Mobile Health App Framework. The authors should address whether any theories are employed to develop the mobile health app framework.

2- Although the authors mentioned that The framework was created following a systematic review of all mHealth apps available on Google Play for Android and App Store for iOS, the manuscript did not address the theoretical framework, which is a fundamental baseline for developing a framework.

Thank you for your comments. Actually, this framework relies on the following:

1. A systematic review of all mHealth apps for epilepsy self-management available on Google Play for Android and the App Store for iOS was conducted to identify the common domains and features of epilepsy self-management. This review was subsequently published

2. A literature review was performed to identify the domains and items of eSM.

3- I could not see the specific processes in the Development of the Development of Epilepsy Self-Management Mobile Health App Framework. The authors mentioned the methodology but not in a sequence.

Thanks. This has been improved as suggested.

4- The manuscript lacks a comprehensive conceptual framework for developing the Epilepsy Self-Management Mobile Health App

5- References are not in line.

This has been fixed.

6. PLOS authors have the option to publish the peer review history of their article ([what does this mean?](https://journals.plos.org/plosone/s/editorial-and-peer-review-process#loc-peer-review-history)). If published, this will include your full peer review and any attached files.


If you choose &ldquo;no&rdquo;, your identity will remain anonymous but your review may still be made public.


**Do you want your identity to be public for this peer review?** For information about this choice, including consent withdrawal, please see our [Privacy Policy](https://www.plos.org/privacy-policy).

Reviewer #1: **Yes:** Esmaeil Mehraeen

Reviewer #2: No

[NOTE: If reviewer comments were submitted as an attachment file, they will be attached to this email and accessible via the submission site. Please log into your account, locate the manuscript record, and check for the action link &quot;View Attachments&quot;. If this link does not appear, there are no attachment files.]

While revising your submission, please upload your figure files to the Preflight Analysis and Conversion Engine (PACE) digital diagnostic tool, <https://pacev2.apexcovantage.com/>. PACE helps ensure that figures meet PLOS requirements. To use PACE, you must first register as a user. Registration is free. Then, login and navigate to the UPLOAD tab, where you will find detailed instructions on how to use the tool. If you encounter any issues or have any questions when using PACE, please email PLOS at [figures@plos.org](mailto:figures@plos.org). Please note that Supporting Information files do not need this step.

<span style="font-style: italic; font-size: 90%;">In compliance with data protection regulations, you may request that we remove your personal registration details at any time. [(Remove my information/details)](https://www.editorialmanager.com/pone/login.asp?a=r). Please contact the publication office if you have any questions.
